# The private sector, international development and NCDs

**DOI:** 10.1186/1744-8603-7-23

**Published:** 2011-07-28

**Authors:** Christine Hancock, Lise Kingo, Olivier Raynaud

**Affiliations:** 1 C3 Collaborating for Health, 1st Floor, 28 Margaret Street, London, W1W 8RZ, UK; 2 Lise Kingo, Novo Nordisk A/S, Novo Allé, DK-2880 Bagsværd, Denmark; 3 Olivier Raynaud, World Economic Forum, 91-93 route de la Capite, CH-1223 Cologny/Geneva, Switzerland

## Abstract

This article addresses an area that has been largely underserved by the development community, and one in which there is a particularly good opportunity for the private sector to take a lead in making a difference to employees, customers and local communities: chronic, non-communicable diseases (NCDs). It highlights the extent of the epidemic of NCDs in developing countries, sets out the 'business case' for the private sector to act on NCDs, and gives examples of initiatives by business to ensure that the healthy choice really is an easier choice for employees, consumers and local communities. It makes the case that, to be genuinely sustainable, businesses should be addressing health as a core part of what they do and, by working in partnership - as called for by the Millennium Development Goals - they can make a real difference and become part of the solution. Identifying ways in which this can be done should form a key part both of planning for, and action after, the UN High-level Meeting on NCDs, to be held in September 2011.

## 1. Introduction: Business - a force for good?

Industry can be a force for real good in developing countries, with the private sector providing jobs and income for employees and their families, goods and services for consumers, and tax revenue and capacity-building opportunities for governments. The impact of business may even be greater than that of governments with their often-limited resources - in an estimate of the 100 largest economies in the world in 2009, 44 were businesses rather than national economies [1]. 'Big business' is often regarded with mistrust, with companies being criticised for focusing solely on profits and failing to take account of the impact of their activities. Whether this takes the form of environmental pollution, the sale of dangerous products, inappropriate marketing or the ill-treatment of workers, this can sometimes imperil its 'licence to operate'. However, the importance of working with responsible businesses to achieve poverty reduction has been acknowledged by the Millennium Development Goals (MDGs), established in 2000, Goal 8 of which calls for a 'global partnership for development', incorporating the private sector, notably (but not exclusively) the pharmaceutical industry and new technologies [2]. Industry can take a role, particularly where the government is not - or cannot - protect the health of its people.

This article focuses on an area that has been largely underserved by the development community, and one in which there is a particularly good opportunity for businesses to take a lead in making a difference to employees, customers and local communities: chronic, non-communicable diseases (NCDs). The four major NCDs - cardiovascular disease, cancers, type 2 diabetes and chronic respiratory disease - are responsible for the majority of the global disease burden. This epidemic has been rapidly escalating in developing countries - and, in recognition of this, the United Nations is to hold a major, High-level Meeting (referred to here as the 'UN Summit') in September 2011, to discuss the issue [3]. There is no time to lose, as NCDs threaten to overwhelm health systems in developed and developing countries alike.

There is a significant precedent for businesses taking a lead in a multi-factor, multi-stakeholder issue: action on the environment. Over the last few years, environmental issues have gone from a niche topic debated by experts and activists, to mainstream debate, and have moved from philanthropy to become part of 'corporate social responsibility'. Decisions on environmental impacts are often now deeply embedded in business models and strategies, with green technologies and processes becoming an important driver of economic growth and opportunity. Health - including NCDs - should be the next wave.

This article sets out the case for action and for the potentially key role that the private sector can and should play in preventing and managing NCDs in developing countries, within its own sphere of influence and by assisting governments in discharging their responsibilities.

In writing this article, the authors have all drawn on their extensive practical experience in this field, as well as knowledge gained through discussion and presentations on prevention and control on NCDs at conferences and meetings with diverse stakeholders.

## 2. The scale of the epidemic of NCDs

The shift in the burden of disease in developing countries from infectious diseases to NCDs has been driven by a number of factors, often indicative of economic development: a move from traditional foods to processed foods high in fat, salt and sugar, a decrease in physical activity as jobs - and lifestyles - become more sedentary, and changed cultural norms (such as increasing numbers of women using tobacco). In addition, there is better access to vaccinations and drugs to prevent and treat infectious diseases, often through aid programmes such as the Global Alliance for Vaccines and Immunisation (GAVI) and the Global Fund to Fight AIDS, Tuberculosis and Malaria. But NCDs have been rising - and will continue to do so: the World Health Organization (WHO) estimates that in Africa, deaths from NCDs will rise by 27 per cent between 2005 and 2015, and that in SE Asia infectious disease deaths will fall by 16 per cent while NCD deaths will rise by 21 per cent [4].

The tragedy is that much of this burden is preventable, through tackling poor diet, tobacco use and lack of physical activity. Diabetes currently affects at least 285 million people around the world, expected to rise to 438 million within 20 years, [5] but at least 50 per cent of type 2 diabetes cases could be prevented or delayed in people with pre-diabetes or with known risks for developing the disease by adopting a healthy diet and increasing physical activity [6]. In addition, many of the 17+ million deaths each year from cardiovascular disease - the world's biggest killer, with 82 per cent of the deaths occurring in low- and middle-income countries [7] - could also be prevented.

Demographic changes have a significant impact on non-communicable diseases - it is a sign of great progress that fewer people die young from infectious diseases - but healthy ageing is increasingly threatened by the rise in NCDs. For example, recent evidence from Canada has shown that the presence of chronic conditions, such as diabetes, has a much greater impact on health-care resources than age alone [8]. A further study in Matlab, Bangladesh, showed what the authors describe as a 'massive change' over a 20-year period (1986 to 2006) in the proportion of deaths from non-communicable diseases compared to infectious diseases - however, there was not a large ageing effect during this time period, so the change was not simply due to increased longevity [9].

In any case, NCDs do not affect only the elderly: many who develop NCDs in developing countries do so at a relatively young age - a recent study of 23 low- and middle-income countries found that 43% of NCD deaths were among those aged under 70 [10]. Where there is very limited access to essential medicines and other treatments, this is likely to impair younger people's ability to work, or remove them (and their carers) from the workforce. It is a terrible irony that, once the poorest are removed from the deepest poverty, lifestyle changes may lead to long-term health problems. In addition, a dual burden of infectious and non-communicable diseases is often faced (and with links between them - for example, diabetes increases the risk of contracting tuberculosis [11]), with different diseases affecting different family members.

That this is a development issue should be self-evident: NCDs affect the incomes of individuals, families and governments. This is ageing without wealth and, as Judith Mackay of the World Lung Federation has put it: 'It is hard to see how the [MDGs] can be achieved without addressing NCDs, especially for the goals related to health outcomes, poverty and hunger.' [12] Economic development is clearly threatened by NCDs - for example, the WHO estimated that the national income foregone in India between 2005 and 2015 due to stroke, heart disease and diabetes would be $236 billion, around 1 per cent of GDP [13].

As one example, spending on one of the major risk factors for NCDs, tobacco (the use of which killed 6 million people in 2010, 72% of whom lived in low- and middle-income countries [14]), can 'crowd out' spending on other essentials such as education and food among the poorest. A 2001 study in Bangladesh, for example, found that the money typically spent by a poor family on tobacco could add 500 calories a day to the diets of one or two undernourished children [15].

And yet, NCDs have not been a priority, either for development agencies or for companies doing business in developing countries. The extent - and speed of transition - of the epidemic of NCDs has not been fully appreciated, and NCDs are often, wrongly, perceived to be 'diseases of affluence', when in fact they often hit the poorest hardest. Infectious diseases such as HIV/AIDS, tuberculosis and malaria, included in the MDGs, have often been prioritised, and great progress has been made. However, working to achieve a more holistic approach to health, working across the health system as a whole, can help to tackle both infectious and non-communicable diseases more effectively than working within disease 'silos' [16].

## 3. Why should business act on NCDs?

Industry has often ignored the negative effect of business activity on national or workplace health, as businesses have not been held economically, politically or morally responsible for the consequences of their actions. This responsibility goes beyond the impacts of environmental pollution and other high-profile health issues: there are also the more insidious impacts of tobacco, sedentary lifestyles, overconsumption of foods high in fat, sugar and salt, and unhealthy workplaces and working practices.

There are many arguments for private-sector action on NCDs. First, there is the profit motive - namely that the drain that NCDs will put on the economy in the coming decades will threaten future profits. This risk is serious: the World Economic Forum's 'Global Risks' project has identified NCDs as being one of the greatest threats to the global economy in 2010, with only risks such as asset price collapse being seen as both more likely and with the potential for greater economic loss [17]. For example, a major epidemic in the developing world would lower worker productivity in BRIC countries (Brazil, Russia, India and China) with repercussions going far beyond the companies operating within those countries, affecting pensions and other investments in the wider economy.

A second reason why the private sector is well placed to tackle NCDs is because businesses often plan for periods much longer than that of the political cycle - and this long timescale is particularly appropriate to addressing the risk factors for NCDs (in contrast to addressing infectious diseases, which require rapid responses, often more conducive to immediate development solutions). Given that the potential rise in ill-health and fall in productivity due to NCDs over the next 20 years is threatening the viability of business-as-usual, industry can develop more sustainable business models that will seek to reduce the major risk factors and promote healthy ageing in the workforce and among consumers, benefiting both the bottom line and the health of the population.

Alone, neither industry nor government - particularly when budgets are stretched - can reverse the growing epidemic: we need to work together. The financial resources, human capacity and market penetration of business can be invaluable in creating partnerships with the public and NGO sectors, helping to create an environment that is health-promoting, in which individuals have the resources and knowledge needed to make healthy choices. This need for partnership was highlighted in the Millennium Development Goals, and specifically by the World Health Organization's 2008-2013 Action Plan for the Global Strategy for the Prevention and Control of Noncommunicable Diseases, [18] which includes as one of its six major objectives the need to 'promote partnerships' in NCDs. Frameworks such as the World Business Council for Sustainable Development are now building on the recognition that collaboration between private and public sectors is needed to address the challenges of poverty, above and beyond the MDGs - and that this includes NCDs [19] - and organisations such as the Pan American Health Organization are also engaging with business (in this case, through its Partners Forum [20]).

By working in partnership, businesses can make a real difference and become part of the solution. Identifying ways in which this can be done should be a key part of planning for the UN Summit in September 2011.

### 3.1 Making the business case

Taking action on NCDs is not merely a 'nice to have' - it is fast becoming an attractive business proposition, an essential part of future-proofing the business that can improve its performance in the short, medium and long term. True sustainability requires the prospect of long-term return, and companies that do not take into account the health of their workforce - and the economic health of local and national communities - will find that the burden of NCDs will damage this return: every company has an interest in engaging in NCD prevention.

The many rationales for considering NCDs in investment decisions can be divided into three clear categories: employees (keeping the workforce healthy), profit motive (new products and new markets) and 'beyond business' (doing the right thing).

#### 3.1.1 Employees

It is in its role as employer that the vested interest of the private sector in tackling NCDs is most obvious. There is increasing evidence, specifically in high-income economies, of the return on investment that can be seen in keeping the workforce well - that an unhealthy workforce makes for an unhealthy business. Particularly where employers are responsible for health-care costs, preventing NCDs through workplace health programmes can have attractive cost-benefit ratios. A healthy workforce is not only more likely to take less time off work through illness (absenteeism): employees are also less prone to 'presenteeism' (under-performing at work), which can be a threat to the competitiveness of the company. Action can be taken on encouraging healthy lifestyles, and also - where necessary - employers can help with provision of early diagnosis and essential medicines.

A meta-evaluation of 56 worksite health promotion economic-return studies in 2005 concluded that workplace health programmes can result in significant reductions in medical and absenteeism costs - of around 25 to 30 per cent - over a 3.6-year timescale, and had an average 5.81:1 ratio of savings to cost [21]. The World Economic Forum is now working, with Professor David Bloom at Harvard University, to analyse more up-to-date data for presentation ahead of the UN Summit in September 2011. Working closely with employees to design programmes - with individual employees taking the role of champions or health advocates within the workplace, and partnering with organised labour - often has the most benefit.

Acting on NCDs is 'the right thing to do' - and, while taking action on, for example, health and healthy lifestyles (even above and beyond what is required to raise productivity) may not seem like the most business-orientated decision, it is likely, in fact, to raise morale and motivation among employees, improve the reputation of the business and increase trust in the company among employees, consumers and government. Where a business acts positively to foster good health, recognising and sharing this will improve trust in the company and provide the business with further incentive to continue to make progress. A survey of over 28,000 people in 15 countries, undertaken in 2009, found a strong correlation between companies that have wellness programmes and companies that a) encourage employees to be creative and innovative and b) that are more likely to retain staff. (While this is not necessarily indicative of a causal relationship, as the report notes, it gives an insight into the levers that companies can use to improve performance, morale and retention.) Respondents who see wellness as a priority for their employer were 3.5 times more likely to report being encouraged to be creative than those for whom it is not a priority - and 64% of them plan to stay for at least five years, compared to only 42% in companies where wellness is not prioritised [22].

#### 3.1.2 Profit motive

All companies have to report to their shareholders, and identify sustainable opportunities to develop new products or reach new markets. The profit motive may be viewed with distrust by those not in the private sector, as profit is not always pursued responsibly - but competitive advantage can be achieved through innovative and responsible approaches, which adapt to local realities. These can lead to greater market share and open up new markets as economic development increases. The challenges of NCDs require new insights into preventing their onset and supporting those who live with the diseases.

The need to adapt to local realities may give companies the opportunity to identify the potential for new, replicable and scalable innovations that will be appropriate not only in the developing country itself, but more widely. Nigel Crisp, in his book *Turning the World Upside Down*, [23] cites the example of Aravind Eye Care (in southern India), which developed new, affordable methodologies and materials to create an intra-ocular lens, costing just US$2 (compared to prices of up to $200 from other manufacturers at the time). The product now has an 8% market share and is used in 120 countries around the world [24].

The development of the polycap (or polypill) - a single daily pill containing a number of generic drugs to prevent NCDs - has also been driven by research and innovation in India, where a number of companies are now manufacturing polypills. The polypills include a statin and several blood-pressure lowering drugs, and the pill for secondary prevention (for people with established disease) also contains aspirin. The potential impact of such a pill could be startling - a report in the *British Medical Journal *in 2003 [25] stated that, were the pill to be taken by everyone over the age of 55, it could reduce ischemic heart disease events by 88% and stroke by 80%. The polypill can be manufactured for as little as $1 a dose and is already available in India - but not in most developed countries, where it and other similar products have yet to be approved. Large-scale studies on its benefits in practice are ongoing.

The NCD epidemic is growing: tackling it, and reversing it (with knock-on effects for communities and national economies) should be part of the sustainable business plan of food, pharma and other industries.

There is a considerable, untapped potential market for 'health', in which all industries - food, media, sports, information technology, insurance - can leverage the fact that health considerations are becoming a major criteria of choice in purchasing decisions.

Current delivery models for healthcare, mostly designed for acute care, are also not adapted to deal with the management of chronic disease, creating the opportunity for healthcare industries to come up with innovative approaches to, for example, early diagnosis, compliance in treatment and remote monitoring.

#### 3.1.3 A 'win-win'?

A recent approach, spearheaded by Michael Porter, highlights the opportunities for business in creating 'shared value' - namely, 'policies and operating practices that enhance the competitiveness of a company which simultaneously advance the economic and social conditions in the communities in which it operates' [26]. Rather than seeing business and society as 'pitted against each other', this approach suggests that there are circumstances in which there can be win-wins for business and for society, partnering to address societal needs and challenges that would otherwise undermine business (such as, for example, the costs of ill health among employees and their families).

As an example of a business and societal win-win, Novo Nordisk in China has pursued a strategy focusing not only on treatment but also supporting physician training and patient education, strengthening the healthcare system, creating public awareness, and establishing local production and R&D. This strategy has exceeded business forecasts - by 2010 Novo Nordisk had a 62% share of the insulin market with sales growth of 30%. As well as being economically sustainable for the company itself, this engagement approach has created significant economic and social value for the Chinese society with 14,600 new jobs in the local economy and 140,000 saved life years [27]. Education of healthcare professionals and patients can also save billions on healthcare budgets. Careful management of diabetes has been shown to lead to considerable savings for Chinese society. While insulin sales per patient will rise (adding US$644 to the cost of insulin over a lifetime), the net saving to society in terms of complications avoided is such that this cost is more than recouped, with a further saving of US$1,820 per patient over a lifetime [28].

### 3.2 Where can business have an influence?

In the past, there has often been reticence within the development community about working with business, despite the calls for partnership in MDG 8. Why would local NGOs, government or other organisations want to take what can be perceived as a risk in working with a business, and why would a business want to take the risk of working with these partners?

The answer is that all businesses have an impact on development and on health - through providing safe jobs and secure income for employees, through the taxes that increase government revenues and allow for greater spending on public goods such as health services - and that joint working by different stakeholders can allow for significantly improved outcomes.

All companies are employers, providers of goods/services, and are participants in local and national communities. These four spheres of influence can help to prioritise what is most important for companies to consider when looking to act in a way that is socially responsible [29].

• **Employees **are closest to home for companies, and are at the heart of what they do: tackling workplace health should be a first priority. This is about moving beyond 'health and safety' and occupational health (which is often simply 'safety' - for which companies are held legally responsible). Employees who are knowledgeable and empowered are not only more productive: they can also be powerful advocates for change within their families and local communities.

• **Consumers or customers**: Some industries - notably the tobacco, food and fitness industries - clearly play a part in the lifestyle choices that can drive or prevent the later development of NCDs. The pharmaceutical industry also has a strong role, as highlighted in the Millennium Development Goals, providing drugs to prevent, control and treat NCDs and their risk factors. Other industries, too - including the media and information technology - are increasingly influential: for example, negative influences such as encouraging overly sedentary lifestyles, or positive influences, such as the development of new tools such as mobile technologies to provide easily accessible, tailored help and information, and remote monitoring.

• **Local communities**: Businesses are often a focal point within the local community, with influence over the wellbeing not only of their employees, but also of their families and other businesses and organisations in the vicinity. These influences can be negative - such as environmental pollution - or positive - such as encouraging use of its health facilities by local community groups.

• **Wider influence**: Finally, large companies, in particular, may have influence at national level, whether through partnering with government, industry-wide alliances or through participating in multi-sectoral action - all of which can support systemic change and foster an environment in which it is easier to be healthy.

Beyond the requirements set by legislation, companies should consider taking the lead 'closest to home' first - ensuring sustainability within the workforce through, for example, setting tobacco-free policies, and carefully considering their responsibility for enabling better health among consumers and local communities, as well as the responsibility not to undermine legislation at national level.

## 4. What can business do to ensure access to health?

Health is defined by the World Health Organization as 'a state of complete physical, mental and social well-being and not merely the absence of disease or infirmity'. 'Access to health', too, goes beyond simple access to health care - it is about empowering people to make choices that enhance physical and mental wellbeing and to act upon those decisions. And industry's role is about much more than just philanthropy or funding - it is about working in partnership to ensure the best health outcomes.

### 4.1 Staying healthy

Access to health begins with retaining good health and preventing disease. This requires that individuals both know what is healthy and have the means to choose the healthy option - both areas in which businesses can have influence.

#### 4.1.1 Awareness-raising and education

Raising awareness of the issues around preventing and managing NCDs in the workplace is a step that companies can take in the short term, for example through providing health information (perhaps through representatives of worker organisations or through trusted health and safety representatives) and by tackling the stigma surrounding NCDs such as diabetes and cancer.

Once people are aware of the issues surrounding NCDs - for example, how to live healthily or identify the symptoms of NCDs - education and training, within and beyond the workplace, can help people to understand what it is that they need to do to tackle the diseases.

Progressive companies can share their education programmes with suppliers and other local companies, perhaps including requiring their suppliers to abide by the same values and programmes on workplace health. There is precedent for health capacity-building in programmes to tackle HIV/AIDS: for example, Eskom, South Africa's main producer of electricity, has been providing advice on wellness programmes, education and voluntary counselling and testing (for HIV/AIDS) to its suppliers, ranging from small- and medium-sized enterprises to large companies, for several years, [30] most recently in a three-year partnership with the South African Business Coalition on HIV and AIDS [31].

A further step that companies can take locally is to supply resources to help to educate health professionals in the community, particularly on NCDs. The Commission on the Education of Health Professionals for the 21^st ^Century has recently found, as reported in *The Lancet*, that health professionals' education does not properly serve populations' needs, often with chronic shortages of doctors, nurses and primary health workers [32] - and an initiative by C3 Collaborating for Health has found a keen interest among nurses in South Africa for more information and assistance with addressing NCDs in local communities [33].

#### 4.1.2 Facilitating healthy lifestyle choices

Awareness and knowledge of how to act to prevent and treat NCDs is not sufficient without a third step: facilitating the necessary lifestyle changes.

This is a particularly fruitful area - all companies can take steps to facilitate lifestyle change within the workplace, and many providers of goods and services can also focus on creating products that encourage consumers to make healthier choices in the community at large, moving well beyond companies' initial sphere of influence in the workplace.

##### a) Making it easier to make the healthy choice at work

The chance that businesses have to promote physical activity at work is highlighted in the WHO's Action Plan, which highlights 'active commuting' and safe, active travel to work. While transport policies and urban planning are the responsibility of government and local authorities, businesses can make it easier for employees by providing facilities such as showers, or allowing breaks of sufficient length to take a walk or other activity. Physical inactivity is the fourth-leading cause of death worldwide [34] and, for example, inactive people can have twice the risk of coronary heart disease compared to the active [35]. As well as preventing diseases such as diabetes and CVD, it can also help to alleviate stress and musculoskeletal conditions, both of which are the cause of significant absenteeism [36].

Providing healthy food options in work canteens is also a way to foster good health among employees. Nike, for example, has helped its supplier company, Tae Kwang Vina (a Korean company, with a major factory in Viet Nam, from which this example is taken), to implement a wide range of initiatives in adopting Nike's code of conduct. This included not only eliminating toxic solvent-based cleaners and glue, but also the provision of a lunch or dinner, based on a nutrition programme (with which the company received technical support). The meal is cooked on-site and consists of rice, soup, meat and vegetables. The company could have implemented the programme half-heartedly - but in fact embraced it, including offering the meals for free, and with real consideration given to dietary balance, including micro- and macronutrients. According to the International Labour Organization, productivity has improved (as a result both of better nutrition and better management), with higher morale and lower absenteeism [37].

Finally, from a western standpoint, in countries where smoking has been banned from public places and where smoking rates have been largely falling (with some notable exceptions in pockets of the population), it is easy to forget that tobacco use is still a huge health hazard, and that the habit is on the increase in many developing countries. There is an opportunity for companies both to educate about the impact of smoking, and to make it easier for employees to choose the healthy option, by adopting smoke-free policies (in countries in which smoking in workplaces is not already banned), and by helping access to smoking-cessation programmes. At the very least, the private sector should support national campaigns, such as on smoke-free legislation - but it is also an opportunity for companies to take the lead in health promotion.

##### b) Developing new technologies

New technologies - particularly the internet and mobile phones - provide a rich source of ways both to disseminate health information and to collect data quickly and easily, from all parts of the world with mobile coverage. They allow for 'crowd-sourcing' - engaging large groups of people to take part in a particular task, such as providing data on disease outbreaks (which can then be verified by more traditional means) - as well as tracking individual data and behaviour [38].

New technologies can also greatly assist health-care workers on the ground. In South Africa, Vodacom (a subsidiary of Vodafone) and medical IT company GeoMed have been piloting a mobile-phone project, Nompilo, since 2009. Nompilo means 'mother of health' in Zulu, and is also the name given to local community care-givers, who visit patients in their own homes. Nompilo allows these care-givers to use mobile phones to send patients' data to the doctors to review at the clinics - this greatly cuts the time spent on filling in daily activity forms (thereby allowing more patient-contact time) as well as educating the care-givers themselves. Other partners in the pilot include three local NGOs [39].

##### c) Facilitating healthy food choices

Population-wide access to affordable, healthy food products is essential in ensuring a good diet - and the food industry has an unparalleled capacity for marketing and promotion that could help to facilitate healthy behaviour change. In recognition of this, [40] and in response to the WHO's 2004 Global Strategy on Diet, Physical Activity and Health, [41] many of the world's largest food companies - General Mills, Kellogg's, Kraft, Mars, Nestlé, PepsiCo, Coca-Cola and Unilever - made five commitments to the WHO's director general, Margaret Chan, in May 2008 [42]. These written commitments cover product composition and availability (including reformulation and fortified foods, new healthy products and portion control), nutrition information (on-pack where possible), appropriate marketing and advertising to children, promotion of physical activity and healthy lifestyles, and partnership (including promoting this approach in 'all markets' and encouraging other companies - including SMEs - to do the same). Where large companies lead, smaller companies can be encouraged to follow suit, potentially moving well beyond what is required by national regulation. Collaboration with the private sector in changing diets for the better has also been explicitly called for in recommendation 8 of the Institute of Medicine's report on *Promoting Cardiovascular Health in the Developing World* [43].

PepsiCo, for example, has begun to deliver on this commitment, [44] launching, in March 2010, a series of commitments on global nutrition criteria across three sectors - products ('provide more food and beverage choices made with wholesome ingredients that contribute to healthier eating and drinking'), marketplace ('encourage people to make informed choices and live healthier') and communities ('actively work with global and local partners to help address global nutrition challenges') [45]. It has set targets including reducing salt, saturated fat and added sugar in key brands, and by 2012 it will 'eliminate the direct sale of full-sugar soft drinks in primary and secondary schools around the globe' and 'display calorie count and key nutrients on our food and beverage packaging'. PepsiCo in the UK, for example, has set a number of health targets in the UK. The scope, boundaries, basis of reporting and independent external assurance is set out in its *Health Report 2010* [46] and the same rigour will be applied to annual updates on progress against these targets.

##### d) Incentives for participation

Incentives can be an innovative way to nudge people into making healthier choices. South African health insurer Discovery is incentivising members of its insurance scheme to take control of their health through its 'Vitality' programme. Vitality members have access to price incentives to make healthy choices, such as discounts on healthy products at Pick 'n' Pay supermarkets (with greater reductions for those who have taken an online health check), and smoking-cessation courses. There are also rewards when members choose the healthy activities, such as reduced costs of flights. Participation in the Vitality programme is voluntary for those with Discovery insurance. Assessment of data of members of the scheme (normalised for NCD status, age, etc.) found that those who are 'highly engaged' in the programme have lower admission rates to hospital in, for example, cardiovascular disease (7.4% lower) and endocrine and metabolic diseases (20.7% lower), as well as shorter stays in hospital and lower costs per patient [47].

Discovery Health has also used another form of incentive - entry into a lottery - in a targeted health-promotion campaign, the 'Right to Know' HIV testing campaign. This was run in 2008-9 by Discovery and the *Sunday Times *(South Africa), and was embraced by many companies - including Shell, Merck and Anglo American - as a way of encouraging their employees to take an HIV test. Discovery Health partnered with two pharmacy chains and negotiated preferential rates for testing, and also encouraged people to go to their GPs for testing, and results were given in person, along with pre- and post-test counselling. Each month, anyone who had the test has a chance of winning a prize of R100,000 (US$14,500). This model could also be used to encourage screening for NCDs and 'know your numbers' on the risk factors (such as cholesterol, BMI and blood pressure).

### 4.2 Beyond health promotion

Companies can do more than promoting the health of employees and consumers: they can also take a role in strengthening health systems for proper prevention and control of NCDs. Monitoring the diseases and risk factors is also required, and partnering with research institutions and others to find new solutions is urgent and necessary.

#### 4.2.1 Managing existing conditions

Many companies already recognise their role in addressing the health crisis in developing countries - most clearly in regions with high prevalence of HIV/AIDS, tuberculosis and malaria. This model could potentially be extended to NCDs. Pharmaceutical companies can improve access to medicine (for example statins, insulin and blood-pressure-lowering drugs) in poor countries, through donations, improving availability, ensuring quality and reducing the costs of their products - and can also work to address NCDs in other ways, going beyond the supply of medicines actually to support their delivery to the populations that need them.

For example, since 2001 Novo Nordisk has been offering insulin at a differential price - a maximum of 20 per cent of the price in Europe, USA, Canada and Japan - to the least-developed countries (as defined by the United Nations). This differential pricing has had a major impact both on increasing access to treatment and to Novo Nordisk sales: in 2010, almost 350,000 people - more than 7% of the people diagnosed with diabetes and an increase of 30% since 2009 - were using Novo Nordisk products in 33 of the least-developed countries; in India, an emerging economy, it is 13%. In Yemen, 34,000 people were being treated with Novo Nordisk insulin in 2010 compared to 3,500 in 2008. In the period during which Novo Nordisk has introduced its differential pricing, insulin has been made more affordable, which has improved access to diabetes care. In Mali, for example, insulin cost 35% of GDP per capita in 2004, but in 2009 it accounted for less than 3% of GDP per capita - and this even assumes a 24% mark-up, as was the case in 2004 [48]. The differential pricing policy ensures that insulin is more available to people even in countries with poor healthcare capacity; however, additional work is being done to improve distribution in collaboration with local businesses, diabetes organisations and ministries of health, including infrastructure development (such as establishing diabetes clinics in public hospitals), provision of diagnostic equipment for diagnosis, monitoring and care, capacity-building, patient and family education as well as advocacy to include diabetes in national plans and priorities, and large-scale awareness-raising initiatives. Private-sector initiatives like these must be followed up with political commitment to break down barriers to access, strengthen public infrastructures and improve the logistics of insulin distribution.

A further way in which companies could help to manage existing conditions is by utilising the extraordinary reach of their distribution channels, often stretching deep into rural populations - soft drinks, for example, are, in many areas, more widely available than many drugs. The UK-based organisation ColaLife is developing an 'AidPod' - a container that fits onto a crate of bottled drinks, which can be filled with essential medicines - and is also creating a sustainable business model that would incentivise the distributors through small payments at each stage. ColaLife is in discussion with local partners in Zambia - including both Coca-Cola and the bottlers SAB Miller - to develop a pilot. The AidPods will initially carry diarrhoea treatment kits - but could in future be developed to contain, for example, the 'polypill' to tackle NCDs [49].

#### 4.2.2 Monitoring

In order to make the most of initiatives to raise awareness, educate, facilitate lifestyle change and manage chronic conditions, companies keen to see the difference that they are making (which will help to make the 'business case'), and to be able to make continuous progress, are well advised to monitor the impact of their programmes - beginning with an understanding of the starting position (gathering baseline data). Monitoring of the workforce, for example, allows the company to assess the benefits of any investment that it is making, and, where personal data is given privately to employees, helps individuals to understand their own health status. It is essential that all health data is gathered and assessed anonymously to protect the privacy of individuals.

Knowledge of the health status of local and national populations can also be useful. This is likely to be reflected in the health of the workforce, and can also allow companies to identify possible future problem areas and take pre-emptive action.

#### 4.2.3 Researching

Research into 'what works' in NCD prevention and management can be supported by industry in partnership with local research institutions. Businesses have great capacity for, and expertise in, market research, and can also provide funding and share their skills to help to build a knowledge base in developing countries. Private-sector involvement in research has been specifically called for in, for example, the Grand Challenges in Chronic Non-communicable Diseases, published in *Nature *in 2007 [50].

UnitedHealth (a major US-based insurance health and wellbeing company) collaborates with the US National Heart, Lung and Blood Institute in supporting a global network of Collaborating Centers of Excellence. Each Center pairs a research institution in a developing country with at least one partner academic institution in a developed country. These Centers of Excellence are developing infrastructures for research and training, enhancing the capacity to conduct population-based or clinical research to monitor, prevent, or control NCDs. For example, the Center in Guatemala City, based at the Institute of Nutrition of Central America and Panama (INCAP), partners with Johns Hopkins University as well as a number of local universities and NGOs, and aims to 'reduce the burden of CVD in Mesoamerica by ... carry[ing] out research, training, and capacity-building for prevention and management of CVD and related risk factors' [51,52].

A further example is the private-sector support of a six-month attachment of communications expertise from GSK that provided a boost to efforts by the Pan American Health Organization to address the burden of NCDs in Latin America and the Caribbean. This strengthened capacity for communications and advocacy related to the prevention and control of NCDs, and added value by bringing a more 'marketing'-orientated view to communications materials.

There is much that remains to be done to tackle the epidemic - and much that we do not yet know about the extent and impact of NCDs and the risk factors. Data-gathering and research into what works are essential for identifying the best use of resources and the most fruitful interventions - but there are already clear indications of where the most fruitful interventions lie and, particularly ahead of the UN Summit, action must be taken now to prevent the burden of NCDs from increasing further over the coming decades.

## 5. Conclusions

The private sector in developing countries - both local companies and international businesses with multinational reach - has great potential for taking a lead in prevention, control and management of NCDs, whether within its immediate sphere of influence (beginning with the health of employees) or in partnership with other organisations and actors in the wider community.

Businesses have many advantages when seeking to address NCDs: they have the geographical presence, the convening power and credibility within local communities, the commitment to be involved over the long term and - crucially - the resources to take action. Other stakeholders - whether government, NGOs, academics or international organisations - can look for and seize opportunities for working in conjunction with businesses to tackle the health challenges facing developing countries.

As Sir George Alleyne, director emeritus of the Pan American Health Organization, has put it: 'Few if any major health programmes with lasting impact globally have been successful without the involvement and active participation of the major social partners - the public sector, the private sector and civil society. Cooperation among them represents our best chance of surviving this tsunami of NCDs.'

## Competing interests

CH is founder and director of C3 Collaborating for Health, a London-based NGO, a registered charity, that works with industry (among other stakeholders) to tackle the prevention of NCDs (all funds received by C3 from businesses and other sources are declared on http://www.c3health.org/aboutc3/); LK is executive vice president and chief of staffs at Novo Nordisk; OR works at the World Economic Forum, a non-profit organisation that works with industry to address the most pressing of the world's problems.

## Authors' information

CH is the founder and director of C3 Collaborating for Health http://www.c3health.org, a small global charity that tackles four major non-communicable diseases (cardiovascular disease, diabetes, chronic respiratory disease and many cancers) by focusing on the three biggest risk factors: tobacco, poor diet and lack of physical activity.

LK is executive vice president and chief of staffs at Novo Nordisk http://www.novonordisk.com, a global healthcare company with 87 years of innovation and leadership in diabetes care. Headquartered in Denmark, Novo Nordisk employs approximately 30,100 employees in 76 countries, and markets products in 179 countries.

OR has been head of the Global Health and Healthcare Sector at the World Economic Forum http://www.weforum.org/ since 2008, before which he spent most of his career at Sanofi Pasteur, a world leader in vaccines, where he held several positions in the International Division, in Europe, Asia (Hong Kong) and Latin America (Argentina), and for the last four years as vice-president in charge of Africa.

## Authors' contributions

CH drafted the manuscript and coordinated its development; LK conceived of the concept for the article, provided case studies and edited the manuscript; OR provided case studies and edited the manuscript. All authors read and approved the final article.
